# The Effectiveness of Drying on Residual Droplets, Microorganisms, and Biofilms in Gastrointestinal Endoscope Reprocessing: A Systematic Review

**DOI:** 10.1155/2021/6615357

**Published:** 2021-04-08

**Authors:** Hefeng Tian, Jiao Sun, Shaoning Guo, Xuanrui Zhu, Han Feng, Yijin Zhuang, Xiu Wang

**Affiliations:** ^1^The First Hospital of Jilin University, Changchun, China; ^2^School of Nursing, Jilin University, No. 965 Xinjiang Street, Changchun, Jilin Province 130021, China

## Abstract

**Background:**

Despite endoscope reprocessing, residual droplets remain in gastrointestinal endoscope working channels. Inadequate drying of gastrointestinal endoscope working channels may promote microbial reproduction and biofilm formation, increasing the risk of infection in patients. This review was designed to provide the current status of gastrointestinal endoscope drying, emphasize the importance of gastrointestinal endoscope drying, and evaluate the effectiveness of different drying methods of gastrointestinal endoscope in reducing residual droplets and microbial growth risk.

**Methods:**

A systematic review was conducted according to the Preferred Reporting Items for Systematic Reviews and Meta-Analyses (PRISMA) reporting checklist. The PubMed, Web of Science, Medline, EMBASE, EBSCO, CNKI, CQVIP, and Wanfang Data databases were searched from 2010 to 2020 to identify eligible articles focused on methods of gastrointestinal endoscope drying and the status of endoscope drying. The following key points were analyzed: type of intervention, amount of residual droplets, major microbial types, and effectiveness of biofilm intervention. JBI quality assessment tool was used to determine bias risk for inclusion in the article.

**Results:**

This review included twelve articles. Two of the articles reported lack of drying of gastrointestinal endoscopes while the other ten reported residual droplets, microbial growth, and biofilm formation after different methods of drying. Four articles reported 0 to 4.55 residual droplets; four articles reported that the main microbial types were *cocci* and *bacilli*, most commonly *Staphylococcus*, *Escherichia coli*, *Bacillus maltophilia*, and *Pseudomonas aeruginosa*; and two reported that drying could effectively reduce biofilm regeneration. The type of intervention is as follows: automatic endoscopy reprocessor (AER), manual compressed air drying, and the Dri-Scope Aid for automatic drying and drying cabinet.

**Conclusions:**

While endoscope reprocessing may not always be effective, an automatic endoscope reprocessor plus the Dri-Scope Aid with automatic drying over 10 min or storage in a drying cabinet for 72 h may be preferred.

## 1. Introduction

Gastrointestinal endoscopy plays an important role in the prevention of digestive diseases [1]. Gastrointestinal endoscopy provides the possibility of early detection of cancer and a strong guarantee for accurate diagnosis of digestive diseases and is of great significance to improve the quality of life of human beings [2]. However, gastrointestinal endoscopes are invasive medical devices with fine structures, complex lumen, and special materials, which makes cleaning and disinfection process difficult. Used endoscopes are used to diagnose and treat subsequent patients, which may lead to endoscope-related cross-infection [3, 4].

The risk of microbial transmission is often underestimated due to the lack of systematic testing and laboratory testing coupled with limited awareness of bacterial transmission among healthcare workers [5]. In the past decades, hospitals around the world have reported endoscopic transmission outbreaks of multidrug resistance and water-borne organisms [6–8]. A striking problem was that some hospitals still had outbreaks despite strict adherence to endoscopic reprocessing guidelines [9]. Previous studies have shown that factors affecting microbial transmission include unreasonable design of instruments, endoscope damage, short cleaning time, low concentration of disinfectants, and inadequate drying [10]. To reduce the spread of endoscopic infection between patients. Endoscopy associations worldwide recommend reprocessing processes that generally include precleaning, leak detection, manual cleaning, rinsing, disinfection, terminal rinsing, drying, and storage [1, 11]. It is worth noting that several recent trials have focused on improving the performance of high-level disinfection (HLD), but few trials have focused on enhancing and assessing the drying process [12–15]. In this paper, the effectiveness of residual droplet, microbe, and biofilm prevention via gastrointestinal endoscope drying is systematically reviewed to provide evidence to improve gastrointestinal endoscope reprocessing guidelines.

## 2. Methods

A systematic review was conducted according to the Preferred Reporting Items for Systematic Reviews and Meta-Analyses (PRISMA) reporting checklist [16] and Joanna Briggs Institute (JBI) literature quality assessment tools [17].

### 2.1. Search Strategy

This systematic review included all prevalence studies and quasirandomized controlled trials related to endoscope drying in the last decade identified from searches in eight databases (PubMed, Web of Science Core Collection, Medline, EMBASE, EBSCO, CNKI, CQVIP, and Wanfang Data) without language restrictions. See Supplementary File 1 for the detailed search strategy. Search terms were first input in each database retrieval box. Next, all the retrieved literature was imported into Endnote for unified management, and then, the titles and abstracts of the articles were independently screened by two reviewers (HF and YJ). If articles did not meet the inclusion criteria, they were excluded. Finally, the screening results of the two reviewers were compared. If there were differences of opinion or disagreement, a third person (XW) was consulted to resolve these issues.

Terms or keywords used in the database included “endoscop∗,” “gastroscop∗,” “gastrointesinal endoscop∗,” “enteroscop∗,” “duodenoscop∗,” “colonoscop∗,” “sigmoidoscop∗,” “proctoscop∗,” “rectoscop∗,” “endoscopic retrograde cholangiopancreatography,” “ERCP,” “dry∗,” and “desiccat∗.”

### 2.2. Eligibility Criteria

In order to make the evidence more adequate and complementary, prevalence studies and quasirandomized controlled trials were selected after screening. The eligibility criteria for these studies were as follows: (1) The type of article could be prospective, retrospective, observational, comparative, or randomized. (2) The contents of the prevalence studies should include the procedure for drying in endoscope reprocessing, and the quasirandomized controlled trials should specify the particular drying intervention methods. (3) The outcome measures for the effects of drying were residual droplets, microbial growth, and biofilm formation to assess the effectiveness of endoscope drying. If a search result only had an abstract without the full-text articles or the drying method was not described in detail, the article was excluded. The case reports of related infections due to insufficient endoscope drying were few and excluded from this systematic review.

### 2.3. Data Extraction and Quality Assessment

Data extraction tables were established in Microsoft Word documents. The two researchers (HF and YJ) independently reviewed all included articles in detail and extracted the relevant data, including the first author, publication date and type of article, type of endoscopy, sample size, measurement tools, intervention or survey modalities, and the results and conclusions of the article. Then, the data were analyzed and summarized, and inconsistent opinions were discussed with a third author (XW) to reach an agreement. Joanna Briggs Institute (JBI) literature quality assessment tools [17] were used to evaluate the prevalence studies and quasiexperimental controlled trials. Joanna Briggs Institute (JBI) literature quality assessment tools are composed of 18 items scored as “1 (yes),” “0 (not clear),” and “-1 (no).” The total quality is categorized as “low,” “moderate,” and “high” [17]. The main items included the intervention, control group, missing visits, data analysis method, sampling method, response rate, and sample size.

### 2.4. Synthesis and Analysis of the Data

Endnote was used to centrally manage the documents, and the data were recorded in Microsoft Word documents for synthesis and analysis. Considering that each article came from different countries, the endoscope reprocessing guidelines were inconsistent, the implementation of endoscope drying methods and outcome measures differed, and the heterogeneity was large, it was impossible to apply meta-analysis techniques to the data in this review. Therefore, this systematic review used descriptive methods to present the findings and results.

## 3. Results

### 3.1. Selection Process

**Figure 1:**
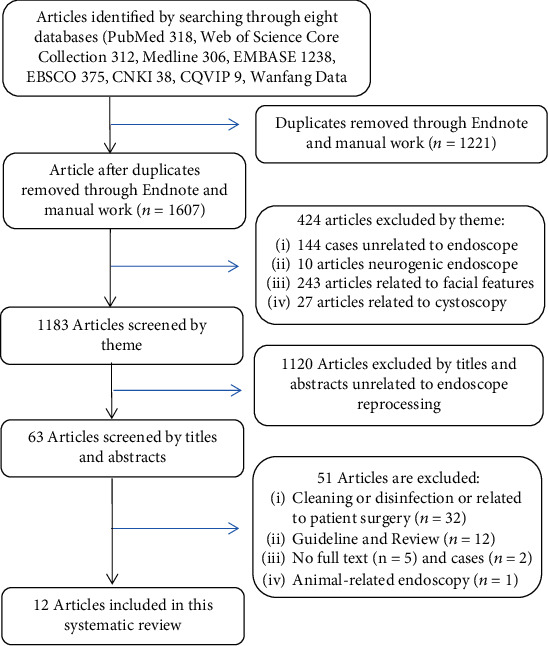
Flow diagram of articles included in the systematic review.

A total of 2828 articles were retrieved from the 8 databases, and 1221 duplicate articles and 424 articles unrelated to the subject were identified using Endnote. The remaining 1183 articles were screened by their titles and abstracts. A total of 1120 articles were excluded according to the eligibility criteria, and the full-text versions of the remaining 63 articles were read. An article from Berlin in 2017 that described an endoscopic reprocessing drying procedure in detail was removed because it reviewed the horse medical endoscopy. Finally, this review included 12 articles. The specific screening flow chart is shown in Figure 1.

### 3.2. Characteristics of the Included Articles

Tables 1 and 2 summarize the characteristics of the included articles, with a total of 12 articles published within ten years. Five of them came from the United States; two came from China; two came from France; and the others were from the Netherlands (*n* = 1), Brazil (*n* = 1), and Ethiopia (*n* = 1). Three of the six prevalence studies reviewed were conducted through questionnaires, and the other three involved direct sampling of hospital endoscopes, with sample sizes from 45 to 295.  Table 3 summarizes the characteristics of the interventions. The six quasirandomized controlled trials were conducted using four drying methods. Two used sterile compressed air drying; two used specific endoscope storage cabinets to achieve medical air or efficient filtration of particulate air drying; one first used 75% alcohol flushing for 3 min then compressed air to enhance the drying effect; and one used Dri-Scope Aid equipment for automatic drying, saving time and manpower. In these included articles, the types of endoscopes involved were gastroscopes, enteroscopes, duodenoscopes, etc. The JBI quality assessment tools were used to determine the risk of bias in the article. Although the twelve articles differed in terms of item bias, they had a low risk of bias overall.

### 3.3. Analysis of the Results

Of the twelve articles reviewed, two articles were related to lack of drying of gastrointestinal endoscopes. The remaining ten articles reported residual droplets (*n* = 4), microbial growth (*n* = 4), and biofilm formation (*n* = 2) after different methods of gastrointestinal endoscope drying. Tables 1 and 2 summarize the intervention and survey methods, results, and conclusions.

#### 3.3.1. Lack of Dryness

Two articles investigated gastrointestinal endoscope dryness [18, 19]. Barbosa et al. [18] evaluated a total of sixty endoscope reprocessing procedures in twenty institutions, and the results showed that there were some deficiencies in the reprocessing procedures; however, those in drying were the most serious. After endoscope cleaning and disinfection, twenty-four (40.0%) endoscopes did not undergo external drying; eighteen (30.0%) were improperly dried; and forty-five (75.0%) endoscope internal working channels were not dried, while only six (10.0%) were dried by compressed air. Another survey used a database of 2026 healthcare workers in the United States to distribute questionnaires to each person via e-mail. A total of 295 questionnaires were received from participants, including 71 physicians and 221 nurses who belonged to 249 different institutions [19]. The data showed that 119 institutions (47.8%) dried by manual flushing of the endoscope channel or incorporating the endoscope into an automatic endoscope reprocessor for compressed air drying, and less than 50% of the investigated institutions used forced medical air drying [19].

#### 3.3.2. Residual Droplets

Table 4 summarizes the effects of various drying methods on residual droplets in endoscopic instrument channels. Four articles reported the presence of residual droplets in endoscopes despite reprocessing [20–23]. Thaker et al. [21] conducted a total of 97 examinations of 59 endoscopes in their hospital. Because no additional compressed air drying was performed before the storage of gastroscopes and colonoscopes, water droplets were observed eight times (35%) during 23 gastrointestinal endoscope examinations. After at least 2 min of additional compressed air drying before storage, no obvious droplets were found. Three endoscopes were vertically stored in a ventilation cabinet for 30 h, and residual droplets were found. After six days of continuous storage, one gastroscope also had obvious moisture [21]. Similar phenomena were also observed in other trials. After 10 min of manual compressed air drying, a small amount of residual droplets was observed in 42.6% (29/68) of endoscope working channels, with a mean ± SD of 0.62 ± 0.95 residual droplets and higher ATP bioluminescence values (*p* < 0.001) [22]. Other researchers [23] investigated 45 reprocessed endoscopes in three American hospitals. After storage for 24 h to 48 h, evidence showed that residual droplets were observed in 21 of 45 (47%) endoscope channels; moisture was detected in 22 (49%) endoscopes; and 10 (22%) endoscope ATP levels were up to 200 RLU. Residual droplets were strongly associated with the level of maximum ATP (*p* < 0.01) [23]. Barakat et al. [20] used three drying methods, including 10 min of manual compressed air drying, 5 min of automatic drying, and 10 minutes of automatic drying. The experimental results showed that 5 endoscope tips observed immediately after manual compressed air drying for 10 min had droplet discharge, with a mean ± SD of 4.55 ± 6.14 droplets; however, after 5 min of automatic drying, only a few droplets were observed, with a mean ± SD of 0.83 ± 1.29 droplets. After 10 min of automatic drying, the average was 0 droplets, and after 72 h of storage, none of the three drying methods resulted in droplets. Whether manual compressed air drying for 10 min significantly differed from automatic drying for 5 min or compared with automatic drying for 10 min, the number of residual droplets was statistically significant, but ATP bioluminescence values were not statistically significant [20].

#### 3.3.3. Microbial Contamination

Four articles mainly explored microbial contamination after gastrointestinal endoscope drying [24–27]. Grandval et al. [24] compared the drying effects of storage cabinets for heat-sensitive endoscopes (SCHE) (all endoscope channels were sequentially rinsed with medical-grade air or highly efficient filtered particulate air (HEPA) to achieve air circulation for 10 min per endoscope and achieve a drying effect) and the drying effects of a conventional storage cabinet, showing that 56.1% (*n* = 23) of endoscopes in the intervention group were not contaminated. After identification, the main microorganisms were *coagulase-negative staphylococci*, *Micrococcus*, and *Bacillus*, and there was no significant difference between the two groups (*p* = 0.829) [24]. Saliou et al. [25] compared the levels of microbes before and after installation of SCHE. The results showed that 45.0% (*n* = 60) of endoscope contamination was detected before installation and only 13.0% (*n* = 69) was detected after installation. There was a significant difference (*p* < 0.001) before and after installation. The identified microbes were mainly *Pseudomonas aeruginosa*, *E. coli*, *Bacillus maltophilia*, pneumococcus, and *Staphylococcus aureus*, and *Candida* was also found in a sample [25]. The effects of drying on *Candida* were evaluated. The results of endoscope sterilization without drying were compared with that of first sterilizing and then drying endoscope channels with compressed air. Evidence demonstrated that 25 Candida strains were isolated at the first evaluation and 9 Candida strains were isolated in the final two evaluations [27]. Chan et al. [26] used 30 ml of 75% ethanol to rinse each channel of the endoscope, let the channels sit for 3 min, and then flushed each channel with compressed air for 30 s; the qualified rate of microbial detection from this method was compared with the qualified rate of microbial detection without drying and compressed air flushing for 30 s. There was a significant difference (A group, 73.73%; B group, 77.08%; C group, 89.52%). Gram-positive bacteria were the most common (mainly *Micrococcus* and *Bacillus subtilis*).

#### 3.3.4. Biofilm Formation

Two articles described the formation of biofilms found in working channels after endoscope drying [28, 29]. Researchers [28] sent questionnaires in envelopes by mail to endoscopy centre in 66 hospitals across the country. Endoscopic channel tubing samples from 66 hospitals were observed by scanning electron microscopy (SEM), and the corresponding endoscope reprocessing procedures were investigated in 66 hospitals. Significant biofilm growth was observed in 36 (36/66, 54.6%) hospitals with 36 endoscope aspiration and biopsy channels and 10 (10/13, 76.9%) water and gas delivery channels. The ratio of alcohol use and air drying in these hospitals was 38.9% (14/36). No biofilms were observed in 76.7% (23/30) of hospitals (*p* = 0.002) [28]. Kovaleva et al. [29] established an endoscope biofilm model in vitro. First, common *Candida* strains were isolated from endoscopes. One of the colonies was inoculated in trypsin soybean broth and incubated at 37°C for 18 h to simulate the formation of endoscope channel biofilms. Disinfection was performed using 1% PAA base disinfectant and then dried using compressed air at 50°C for 2 h to simulate the disinfection and drying process of endoscope reprocessing, and the endoscope was finally stored in a drying cabinet for 7 days. The results showed that the biofilm regenerated after disinfectant treatment without drying, but after drying at 50°C for 2 h or at room temperature for 1, 3, or 5 days, there was no biofilm regeneration [29].

## 4. Discussion

To the best of our knowledge, this is the first systematic review that summarizes the current status of gastrointestinal endoscope drying methods and assesses their effectiveness in preventing residual droplets, microorganisms, and biofilms. This review included a total of 12 articles, with approximately 345 institutions and 783 endoscopes in 958 examinations, which allowed important conclusions on potential advantages and limitations during gastrointestinal endoscope drying. This review showed that the extent of endoscopic drying was not sufficient in clinical practice, as recommended in existing guidelines for gastrointestinal endoscope reprocessing; moreover, the guidelines or statements for endoscope reprocessing around the world are inconsistent, and no clear and uniform requirements have been made on the duration and method of drying. Gastrointestinal endoscopes still had residual droplets, microbial growth, and biofilm formation after reprocessing and drying, but we found that automatic endoscope reprocessor (AER) plus Dri-Scope Aid automatic drying was obviously better than AER plus manual drying and the use of drying cabinet once again reduced the residue of droplets and the growth of microorganisms, thus improving the quality of gastrointestinal endoscope reprocessing.

Drying procedure is an important factor affecting the quality of gastrointestinal endoscope reprocessing and should not be underestimated [30, 31], and the drying method is a vital means to improve the drying effect [32]. Insufficient endoscope drying allows microbes to easily breed, causing infections in patients. Five studies in France reported the isolation of *Bacillus pneumoniae* and multidrug-resistant *Pseudomonas aeruginosa* from duodenoscopes and gastroscopes used in patients. An examination of the endoscope reprocessing procedures revealed that the cleaning and drying times were too short to adequately comply with endoscope reprocessing guidelines [30, 33–36]. In order to standardize the procedure of gastrointestinal endoscope reprocessing, some national gastrointestinal endoscopy societies have developed guidelines for gastrointestinal endoscope reprocessing. Although each procedure of the guidelines for endoscopic reprocessing varies slightly between countries, there is not much difference overall. Because of the lack of strict regulation and supervision of the best methods for drying of endoscopes after HLD, it is not novel that the actual drying practices vary greatly between institutions, even if the same medical system is used [19, 22, 23]. In summary, endoscope drying can be performed with an automatic endoscope reprocessor, which involves cleaning, a high level of disinfection, and one minute of air flushing; rinsing first with 70% to 90% ethyl or isopropyl alcohol and then manual drying using a safety air gun with compressed air for 30 s or 10 min; and AER plus Dri-Scope Aid for automatic drying for 5 min or 10 min or in a drying/traditional storage cabinet [20, 24–26].

According to the Australian Infection Control in Endoscopy Consensus Statements [37], Korean Society of Gastrointestinal Endoscopy Guidelines [38], Multisociety guideline on reprocessing flexible GI endoscopes [39], American Society for Gastrointestinal Endoscopy (ASGE) [40], Association for Professionals in Infection Control (APIC) [41], Japan Gastroenterological Endoscopy Society (JGEC) [42], World Gastroenterology Organization/World Endoscopy Organization (WGO/WEO) Global Guidelines [43, 44], Canada Infection Prevention and Control Guideline [45], and Society of Gastroenterology Nurses and Associates [46], 70% to 90% alcohol should be used to flush the endoscope working channel before storage, followed by thorough compressed air drying (AER or AER plus manual drying). Alcohol flushing can not only accelerate the drying process but also inhibit the possible existence of water microorganisms. However, the British Society of Gastroenterology Endoscopy Guidelines (BSGES) for decontamination of equipment for gastrointestinal endoscopy [47, 48] and European Society of Gastrointestinal Endoscopy (ESGE) guideline [11] highlight that its use is not recommended because alcohol has potentially fixed effects and unknown risks. Some studies [26, 49] revealed that alcohol flushing can effectively reduce the chance of residual droplets and microbial growth in endoscope channels. Our comprehensive analysis also showed that alcohol facilitated drying of endoscope channels and its use was not found to endanger the health of others.

Our results indicated that AER plus Dri-Scope Aid for automatic drying for 5 min or 10 min was significantly better than AER or AER plus manual drying in preventing residual droplets and microbial growth. While the Dri-Scope Aid for automatic drying was not mentioned in the gastrointestinal endoscopy guidelines, a recent trial suggested that the Dri-Scope Aid for automatic drying for 10 min seemed to greatly reduce the risk of microbial contamination and droplet residue [20]. The Dri-Scope Aid device provides an automatic method to manage forced air in programming time and completes the drying of all endoscope channels, which effectively avoids the mistakes caused by manual drying [20]. The Centers for Disease Control [50] also placed special emphasis on automation of reprocessing to “reduce the likelihood that the necessary reprocessing steps will be skipped.” However, the evidence strongly supporting similar conclusions about endoscopic drying is rather limited, with only one trial [20]. Whether AER plus Dri-Scope Aid for automatic drying can efficaciously prevent droplet residue and microbial growth needs to be further verified by more high-quality clinical trials.

In addition, our review also showed that the use of drying cabinets can reduce the risk of microbial contamination. As guided by many guidelines [47, 48, 51, 52], endoscopes should be stored in a drying cabinet with a drying system that circulates and forces sterile, dry, and filtered air through the endoscope channels. Compressed filtered air can minimize the risk of microbial contamination of cabinets and stored endoscopy. The guidelines do not specify how long endoscopes are stored in the drying cabinet to achieve adequate drying. Several experiments [24, 25, 53] revealed that endoscopes were dried and stored in a drying cabinet for 72 hours, making the results of endoscope microbial quality satisfactory.

As no single drying method has been shown to be valid to achieve endoscope channels without droplet residue and microbial growth, one may speculate that multiple times, combined drying methods based on a case-by-case decision may have the potential to affect the final endoscope reprocessing quality. In fact, this is the key information of a recent trial, where implementation of the process of twice endoscopic reprocessing resulted in significant improvements about the residue in the endoscope [54]. In clinical practice, it is best to recommend using the Dri-Scope Aid to dry for more than 10 minutes or to store in the drying cabinet for 24 hours, and if necessary, to carry out two cycles of reprocessing, which can minimize fluid residues and microbial contamination in endoscope channels and avoid cross-infection between patients, thus providing some reference significance for the later updating of the guidelines for gastrointestinal endoscopy reprocessing. In the future, we will further research a combined drying method, including the use of an automatic endoscope reprocessing machine for initial drying, followed by the use of Dri-Scope Aid for final drying and eventually stored in a drying cabinet to explore the optimal drying method and drying time.

## 5. Limitations

There were several potential limitations in this systematic review. We included quasirandomized controlled trials and prevalence studies. The selection criteria and grouping processes for samples from these studies differed. Moreover, the heterogeneity was large mainly because there were different drying methods, including manual drying, automatic drying, and drying cabinets. The effectiveness of automatic drying was well demonstrated. Most studies did not adequately report the process of gastrointestinal endoscopic drying, limiting further analysis of the details of the drying procedure.

## 6. Conclusions

Accurate drying and storage procedures are important for maintaining bacteria-free endoscopes. Many national guidelines have repeatedly emphasized that effective drying of the internal and external surfaces of endoscopes was as important as effective cleaning, disinfection, or sterilization and that endoscopic equipment must be thoroughly dried before storage to prevent microbial reproduction [1, 11, 38, 51, 55, 56]. However, there are few prevalence studies and quasirandomized controlled trials on the current status of gastrointestinal endoscope drying. Therefore, whether existing drying methods can thoroughly dry endoscope working channels requires further verification. This systematic review reports that endoscope drying practices may not always effectively remove residual droplets, microorganisms, and biofilms in endoscopes, but existing evidence suggests that automatic drying may be superior to other drying methods, drying for more than 10 min or storing in drying cabinets for more than 72 h, which highlights the importance of strict adherence to drying guidelines to make drying procedures more standardized and automated. In addition, the implementation of multicentre, large-sample, and high-quality studies to compare the effectiveness of different drying methods for droplets, microorganisms, and biofilms in endoscope working channels is necessary.

## Figures and Tables

**Table 1:** Characteristics of the quasi-randomized controlled trials.

First author (year)	Country	Study design	Intervention methods	Sample	Outcomes and measurement	Conclusions
Kovaleva (2010) [29]	Netherlands	Quasirandomized controlled trial	(a) PAA-based disinfectant with additional sterile compressed air(SCA) (b) PAA-based disinfectant without additional drying	Biofilm in endoscopes	MTT signal, colony-forming units	PAA-based disinfectant with SCA reliably removed biofilm additional
Grandval (2013) [24]	France	Quasirandomized controlled trial	(a) Storage cabinets for heat-sensitive endoscopes (SCHE) (b) Clean, dry, dedicated cupboard without morning disinfection (c) Clean, dry, dedicated cupboard with morning disinfection	Endoscopes (*n* = 41)	Endoscope contamination levels	SCHE might maintain the microbiological quality of endoscopes
Saliou (2015) [25]	France	Quasirandomized controlled trial	(a) In a clean, dry, dedicated cupboard (b) In an AS300 Hysis SCHE	(a) *n* = 60 (b) *n* = 69	Endoscope contamination levels	The use of SCHE significantly reduced the rate of contaminated endoscopes
Hassaine-Lahfa (2017) [27]	Algeria	Quasirandomized controlled trial	(a) Without drying the material after sterilization (b) Drying the endoscope's channels with compressed air after sterilization	(a) *n* = 100 (b) *n* = 200	The number of Candida sp. isolates	The effects of drying by compressed air enhanced their sterilization
Wong Chan (2017) [26]	China	Quasirandomized controlled trial	(a) Without drying (b) Dried with compressed air (c) With 75% alcohol then dried with compressed air	(a) *n* = 99 (b) *n* = 96 (c) *n* = 105	Colony-forming units; detection rates of pathogenic microorganism	75% alcohol plus compressed air enhanced their sterilization and was superior to compressed air
Barakat (2019) [20]	America	Quasirandomized controlled trial	(a) Manual forced air drying (b) Automated drying for 5 min (c)Automated drying for 10 min	Endoscopes (*n* = 23)	Retained fluid; ATP bioluminescence values	Automated drying may decrease the risk of endoscope infection transmission

MTT: measuring the tetrazolium salt; PAA: peracetic acid-based; SCHE: storage cabinet for heat-sensitive endoscopes; ATP: adenosine triphosphate bioluminescence. SEM: scanning electron microscopy.

**Table 2:** Characteristics of the prevalence studies.

First author (year)	Country	Study design	Survey methods	Sample	Outcomes and measurement	Conclusions
Barbosa (2020) [18]	Brazil	Prevalence study	Data in a checklist were collected by direct observation	Endoscopes (*n* = 60)	Endoscope reprocessing procedures	Failure in many different reprocessing steps: prewash, chemical, and mechanical cleaning, and the rinsing and drying of the endoscopes
Ren-Pei (2014) [28]	China	Prevalence study	The questionnaire was sent to 66 hospitals to investigate reprocessing procedures for endoscopes	Endoscopes (*n* = 66)	Endoscope channel tubing samples were observed by scanning electron microscopy (SEM)	The formation of endoscopic biofilm may be related to reuse of detergent, manual cleaning, and incomplete drying
Barakat (2018) [22]	America	Prevalence study	A total of 85 inspections were performed on all 68 endoscopes in our endoscopy unit	Endoscopes (*n* = 68)	Endoscope working channels were examined by the SteriCam	Residual fluid in our study was most commonly noted with in the first 24 hours after reprocessing, some for up to 72 hours
Ofstead (2018) [23]	America	Prevalence study	Researchers conducted field surveys of fully reprocessed endoscopes. Data were collected during site visits	Endoscopes (*n* = 45)	Endoscopes were stored for 24-48 h before visual examinations; retained fluid was photographed with a camera and borescopes	Inadequate reprocessing and drying contributed to retained fluid and contamination found in a multisite study
Thaker (2018) [19]	America	Prevalence study	A pilot inspection study using a prototype borescope was performed on routinely used endoscopes after HLD, manual forced-air dry of the instrument channel	Endoscopes (*n* = 59)	Video recordings were reviewed for visible moisture, debris, discoloration, scratches, channel shredding, and visible evidence of biofilm	Manual forced-air drying of the channel appears to be highly effective in eliminating moisture compared with overnight hang drying alone
Thaker (2018) [21]	America	Prevalence study	The survey was sent electronically to providers who potentially performed or participated in ERCP in the United States	Institutions (*n* = 249)	Reprocessing techniques, barriers to ethylene oxide sterilization, microbial testing, reprocessing opinions	Improved adherence to forced-air drying in duodenoscope reprocessing is needed

SEM: scanning electron microscopy; HLD: high-level disinfection; ERCP: endoscopic retrograde cholangiopancreatography.

**Table 3:** Characteristics of the interventions.

First author	Year	Intervention description	Duration
Kovaleva	(2010)	(a) PAA-based disinfectant with addition sterile compressed air (SCA) (b) PAA-based disinfectant without additional sterile compressed air	(a) Using SCA for 2 h at 50°C. The two interventions were stored in drying cabinets for 1, 3, 5, and 7 days
Grandval	(2013)	(a) AEWD then storage cabinets for heat-sensitive endoscopes (SCHE) (b) AEWD then in a clean, dry, dedicated cupboard without morning disinfection (c) AEWD then in a clean, dry, dedicated cupboard with morning disinfection	Endoscopes of the three interventions were stored for 72 h then sampled
Saliou	(2015)	(a) AEWD then in a clean, dry, dedicated cupboard (b) AEWD then in an AS300 Hysis SCHE	(a) High-level disinfection after 12 h of storage as recommended in France (b) All channels of the endoscopes were purged with medical air for 1 hour
Hassaine-Lahfa	(2017)	(a) Without drying the material after sterilization (b) Drying the endoscope's channels with compressed air after sterilization	Endoscopes of the two interventions were immersed in Hexanios for 20 minutes and Steranios 2% for 15 minutes
Wong Chan	(2017)	(a) Without drying (b) Dried with compressed air (c) With 75% alcohol then dried with compressed air	(a) Dried with compressed air for 30 s (b) With 75% alcohol for 3 min then dried with compressed air for 30 s
Barakat	(2019)	(a) AER plus manual forced air drying (b) AER plus automated drying for 5 min (c) AER plus automated drying for 10 min	(a) Manual drying of the endoscope working channel for 10 minutes with forced high-efficiency particulate filtered air (HEPA) (b) and (c) Endoscopes were attached to the Dri-Scope Aid device for either 5 minutes or 10 minutes

PAA: peracetic acid-based; SCA: sterile compressed air; AEWD: automatic endoscope washer-disinfector; SCHE: storage cabinets for heat-sensitive endoscopes; AER: automated endoscope reprocessor; HEPA: high-efficiency particulate air.

**Table 4:** Evaluation of the effects of different drying methods on residual fluid droplets in endoscopic working channels.

First author	Drying methods	Endoscopy of droplets (*n*)	Total endoscopy (*n*_1_)	*n*/*n*_1_ (%)	≤30 min droplets	24 h droplets	48 h droplets	≥72 h droplets
Barakat	AER (alcohol flushing and 1 min air flushing) and manual forced air drying for 10 min	5	5	100	4.55 (6.14)	1.62 (1.61)	0.51 (0.7)	0 (0)
Barakat	AER (alcohol flushing and 1 min air flushing) and automated drying for 5 min	4	5	80	0.83 (1.29)	0.20 (0.34)	0.04 (0.11)	0 (0)
Barakat	AER (alcohol flushing and 1 min air flushing) and automated drying for 10 min	0	5	0	0 (0)	0.01 (0.07)	0 (0)	0 (0)
Ofstead	AER (alcohol flushing and 1 min air flushing) and medical-grade forced air drying for 10 min	21	45	47	Not done	a few	a few	Not done
Barakat	AER (alcohol flushing and 1 min air flushing) and manual forced air drying for 10 min	29	68	42.60	0.62 (0.95)	Not done	Not done	Not done
Thaker	Manual forced-air drying at room temperature. Medical air for more than 2 min in each port	8	97	8.2	Not done	A few	Not done	Rare

AER: automated endoscope reprocessor.

## Supplementary Materials

Supplementary Materials Supplementary file 1: search strategy.
