# Supplementary material for: The Effectiveness of Drying on Residual Droplets, Microorganisms, and Biofilms in Gastrointestinal Endoscope Reprocessing: A Systematic Review
Source: Gastroenterol Res Pract. 2021 Apr 8;2021:6615357. doi: 10.1155/2021/6615357 (PMC8049816; doi:10.1155/2021/6615357)
Supplement: Supplementary Materials — Supplementary file 1: search strategy. [file 6615357.f1.docx]

Supplementary file 1. Search strategy

Database Search terms Number of articles

(Ten years)

PUBMED endoscop* or gastroscop* or gastrointesinal endoscop* or enteroscop* or duodenoscop* or colonoscop* or sigmoidoscop* or proctoscop*

318

312

306

1236

375

232

9

38

内镜 and 干燥

内镜 and 干燥

内镜 and 干燥

or rectoscop* or endoscopic retrograde cholangiopancreatography or ERCP and (dry* or desiccat*) not bronchoscop* not laparoscop*

Web of Science ((endoscop* or gastroscop* or (gastrointesinal endoscop*) or enteroscop* or duodenoscop* or colonoscop* or sigmoidoscop* or (ERCP)

or proctoscop* or rectoscop* or (endoscopic retrograde cholangiopancreatography)) and dry* not (bronchoscop* not laparoscop*))

MEDLINE ((endoscop* or gastroscop* or (gastrointesinal endoscop*) or enteroscop* or duodenoscop* or colonoscop* or sigmoidoscop* or (ERCP)

or proctoscop* or rectoscop* or (endoscopic retrograde cholangiopancreatography) ) and dry* not bronchoscop* not laparoscop*)

EMBASE (endoscop* or gastroscop* or 'gastrointesinal endoscop*' or enteroscop* OR duodenoscop* or colonoscop* or sigmoidoscop* or (ERCP)

proctoscop* or rectoscop* or 'endoscopic retrograde cholangiopancreatography') and dry* not bronchoscop* not laparoscop*

EBSCO host (endoscop* or gastroscop* or (gastrointesinal endoscop*) or enteroscop* or duodenoscop* or colonoscop* or sigmoidoscop* or (ERCP)

proctoscop* or rectoscop* or (endoscopic retrograde cholangiopancreatography) or (ERCP) and dry* not (bronchoscop* and laparoscop*))

WANFANG DATA

CQVIP

CNKI
